# 

**Published:** 1910-11-26

**Authors:** 


### CONTENTS.

| | PAGE |
|---|---|
| **A Study in Economic Medicine** ... ... ... ... | 245 |
| **Medical Reasons for Divorce** ... ... ... ... ... | 246 |
| **Annotations**— | |
| The Wandsworth Inquest—The Nobel Prize in Medicine—The American Aviation Accident—"The Child" ... ... | 247 |
| **Medical Opinion and Movement** ... ... ... ... | 248 |
| **Hospital Clinics**— | |
| Demonstration of Cases of Nervous Disease. By Purves Stewart, M.D., F.R.C.P. ... ... ... ... ... ... | 251 |
| **Medicine**— | |
| The Physical Signs of Enlargement of the Gall-Bladder ... | 253 |
| Coma Due to Acute Yellow Atrophy of the Liver ... ... | 254 |
| **Ophthalmology**— | |
| After-Results of Magnet Extraction... ... ... ... ... | 255 |
| **Therapeutics**— | |
| Some Prescription Difficulties... ... ... ... ... ... | 255 |
| **News and Events of the Week** ... ... ... ... | 257 |
| **Obituary** ... ... ... ... ... ... ... ... ... | 258 |
| **Parliament and Publications** ... ... ... ... ... | 258 |
| **The Week's Work**— | |
| Reform of Charities' Help in Australia A Children's Hospital for Leeds—Disinfection of Floors in L.C.C. Schools—Hospital Legislation in Victoria—A Model Hospital Report—Hospital Advertisement; the Wrong Way—A Children's Fund for Middlesex This Week's Drug Market ... ... ... ... | 260 |
| **Special Institutional Articles**— | |
| An American View of State Aid for Hospitals ... ... ... | 261 |
| Public Grants for Voluntary Hospitals ... ... ... ... | 264 |
| Hospital Medical Schools and the King's Fund ... ... ... | 265 |
| The Care of the Feeble-Minded at Leicester ... ... ... | 267 |
| The State Children's Association ... ... ... ... ... | 267 |
| **Gifts and Bequests** ... ... ... ... ... ... ... | 268 |
| **Institutional Notes and News**... ... ... ... ... | 269 |
| **The Hospital World** ... ... ... ... ... ... ... | 271 |
| **Appointments and Resignations** ... ... ... ... | 272 |
| **Jottings** ... ... ... ... ... ... ... ... ... | 273 |
| **New Appliances and Things Medical** ... ... ... | 274 |

